# Bag-of-words is competitive with sum-of-embeddings language-inspired representations on protein inference

**DOI:** 10.1371/journal.pone.0325531

**Published:** 2025-08-06

**Authors:** Frixos Papadopoulos, Tilman Sanchez-Elsner, Mahesan Niranjan, Ashley I. Heinson

**Affiliations:** 1 Vision-Learning-Control Group, Department of Electronics and Computer Science, Faculty of Engineering and Physical Sciences, University of Southampton, Southampton, United Kingdom; 2 Clinical and Experimental Sciences, Department of Medicine, University of Southampton, Southampton, United Kingdom; 3 Clinical Informatics Research Unit, Cancer Sciences, Department of Medicine, University of Southampton, Southampton, United Kingdom; University of Portsmouth, UNITED KINGDOM OF GREAT BRITAIN AND NORTHERN IRELAND

## Abstract

Inferring protein function is a fundamental and long-standing problem in biology. Laboratory experiments in this field are often expensive, and therefore large-scale computational protein inference from readily available amino acid sequences is needed to understand in more detail the mechanisms underlying biological processes in living organisms. Recently, studies have utilised mathematical ideas from natural language processing and self-supervised learning, to derive features based on protein sequence information. In the area of language modelling, it has been shown that learnt representations from self-supervised pre-training can capture the semantic information of words well for downstream applications. In this study, we tested the ability of sequence-based protein representations learnt using self-supervised pre-training on a large protein database, on multiple protein inference tasks. We show that simple baseline representations in the form of bag-of-words histograms perform better than those based on self-supervised learning, on sequence similarity and protein inference tasks. By feature selection we show that the top discriminant features help bag-of-words capture important information for data-driven function prediction. These findings could have important implications for self-supervised learning models on protein sequences, and might encourage the consideration of alternative pre-training schemes for learning representations that capture more meaningful biological information from the sequence alone.

## Introduction

Protein sequences capture significant information about how proteins work and subsequently about the functions of cells and living organisms [1]. Within the last decade, the number of known protein sequences in databases has increased 10-fold from three million [2] to roughly 52 million [3]. However, the number of sequences with annotated functions is much lower, as characterising protein properties experimentally is a challenging and resource-intensive task [4]. Considering that the raw sequence determines many protein properties [1], the ability to perform computational annotation of proteins from the readily available sequence data is important and can lead to an improved understanding of complex biological processes and disease-causing mechanisms.

Earlier computational sequence-based methods for protein inference were based on statistical sequence-alignment approaches [5]. Such methods utilise knowledge of evolutionary and biochemical characteristics of proteins, which has the potential advantage of making the prediction process more interpretable. However, alignment-based methods tend to be highly computationally resource-intensive even with the use of heuristics, especially with the current exponential growth of protein sequence databases. Additionally, they often fail to accurately predict function for sequences that have less than 30% identity with any protein in the database used for querying (“twilight” zone of alignment-based methods) [5]. Furthermore, functional annotation transfer from high-quality databases such as the Gene Ontology (GO) hierarchy [6] does not necessarily become more effective when the annotations of GO terms increase. Because of high sparsity in the distribution of annotations roughly half of the annotation terms are only associated with one gene and, also are not sufficiently informative as they are found in shallow hierarchy nodes [4]. These limitations of alignment-based methods suggest that a benefit could be realised by implementing machine learning approaches to perform protein inference. Importantly, the way in which the protein sequence is represented mathematically is key for the success of downstream machine learning prediction models. Even the best machine learning algorithms display lower performance compared to simpler ones when the representations consist of irrelevant features, whereas generally less capable algorithms produce good results when quality representations are provided as input data [7–9].

Natural Language Processing (NLP) has seen several new Self-Supervised Learning (SSL) algorithms [5]. In SSL with NLP, it has been noted that sentences in a language are arranged in a meaningful way, and the context in which words of a sentence appear in can carry useful information for learning data representations. This is called the “distributional hypothesis” [5,10] and is the underlying idea behind the word2vec algorithm [11], which maps words as symbolic tokens to continuous-valued distributed representations. Based on this hypothesis, word2vec encodes commonly co-occurring words together in the resultant embedding space, reflecting the varying degrees of similarity that words can have [11,12]. Word2vec is thought to lessen some of the issues of earlier word representations, by reducing the dimensions of a vocabulary-size embedding space to dense representations of 100-300 dimensions [13]. Word2vec embeddings can capture syntactic and semantic information of words fairly well to enable the development of downstream models in NLP [12,14].

More recently, a more powerful approach has been built on top of advances in neural language translation for modelling long-range sequences, namely transformer models [5,14]. The key ingredient of the success of transformers has been considered to be the attention mechanism, which enables modelling of long-range dependencies across the whole input sequence, enhancing the ability to capture meaningful relationships within the input features. By learning positional embeddings for each word, the resulting output word embeddings are able to keep ordering and context information around the word. On top of that, the computations involved with attention can be parallelised leading to faster training, but on the expense of higher memory requirements. The pre-training task of the model is in most cases a version of the masked-language-modelling (MLM) cloze task [15], which involves predicting missing word(s) in the input given the context around it similarly to word2vec Continuous Bag-Of-Words (CBOW) architecture [11]. Transformers have been the main idea behind lots of recent algorithms reaching state-of-the-art performance in question-answering, text summarisation, language translation etc. [14,16].

The word2vec model has been re-purposed to build sequence-based representations of proteins in the ProtVec study [17]. To model the language of life, amino acid trigrams are the “words” for which embeddings are built and their sum can be used to represent the whole protein sequence (the “sentence”). These protein representations yielded effective results in protein family (93%) and disordered protein classification (99%) problems [17]. Encouraged by this, several communities have developed distributed vector representations in their application domains, such as dna2vec [18], mol2vec [19] and node2vec [20,21]. ProtVec has been widely adopted in the space of protein inference [8,22], with studies on: predicting protein Glycation sites [23]; modelling protein-protein interaction binding sites [24,25]; improving compound-protein interaction inference [26]; discovering nuclear targeting signal sequences [27]; inferring MHC binding [28]; predicting protein solubility [29]; predicting SARS-COV-2 evolution/mutations [30]; predicting antifungal peptides [31]; classifying anticancer peptides [32]; inferring anti-inflammatory peptides [33]; and finally antiviral peptides [34].

As a natural successor to ProtVec, the ProtTrans study has applied a variety of transformer architectures to test their usefulness under a transfer-learning setting for protein inference [35]. These approaches are able to model the entire input protein sequence at once using the attention function, and learn the mapping of evolutionary patterns present across the sequence sub-units much better. Thereinto, various transformer models inspired from language processing were re-purposed for building protein representations and evaluated on problems such as 2-class sub-cellular localisation and 3-class secondary structure prediction with particular success (reaching accuracies >80% in both) [35]. By innovations such as the attention mechanism for modelling longer-range sequence dependencies and positional embeddings to model temporal amino acid order, versions of the ProtTrans transformer models have shown quite impressive abilities in encoding the important biological information to help improve performance in several inference problems [31,33,36–40].

Inspired by the aforementioned work, in this study we focus on the hypothesis: to what extent can pre-trained language-based representations capture properties of proteins? To quantify this, we first compare ProtVec representations to a baseline method based on the Bag-of-Words (BoW) approach often used in NLP [13]. BoW is considered a naive approach that builds histograms of word counts for each sentence in the data. It is thought to suffer from high-dimensionality and implicitly considers words as unrelated tokens, both shortcomings which word2vec (that is the backbone of ProtVec) is expected to overcome. We test these two representation methods on twelve protein inference problems including function and structure-adjacent tasks. Following this, we also compare BoW to the ProtT5 representation [35] across seven of the function inference problems. Summarizing, our main contributions are:

- This is the first study that systematically tests the performance of self-supervised learned representations against simpler baseline approaches such as Bag-of-Words histograms on a variety of protein inference tasks, including large amounts of microbial data.
- By feature selection we show that the top discriminant AA trigrams help bag-of-words capture important region-specific information for efficient data-driven function prediction.
- We speculate that the intriguing results of our comparisons would motivate the community to critically re-consider several language model design choices to create more suitable representations for protein inference problems. Future directions could focus on injecting biological sequence priors during the pre-training representation learning step.

The rest of this article is organised as follows: Following the Introduction we present the ‘Materials and methods’ with more details on the experimental setup, representations and datasets used for the protein inference problems in this study; Then the results section is structured with five sub-sections, each focusing on a specific contribution; Finally, we discuss the significance of our contributions.

## Materials and methods

### Protein representation methods

**Fig 1:**
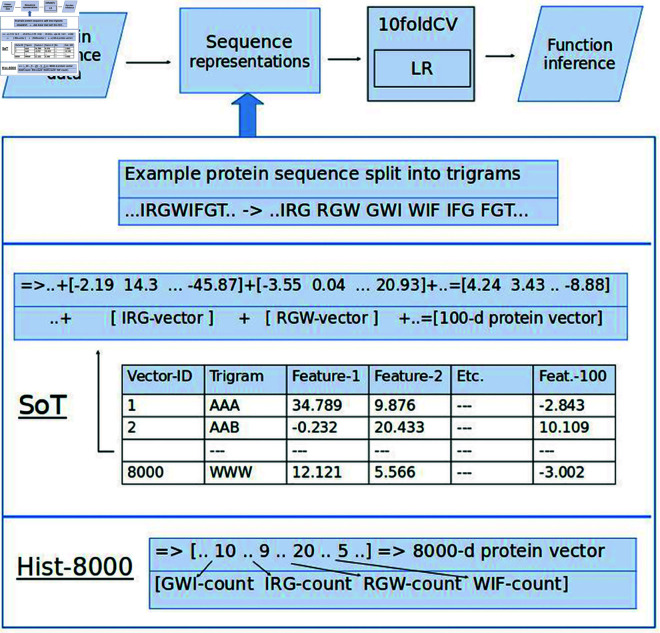
Main classification setup and protein representation methods. LR is evaluated under 10foldCV using the AUC metric for binary classification. The SoT method assigns each trigram in the split sequence to the corresponding embedding obtained from SSL pre-training [17], and then sums up the embeddings to get a 100-d representation. Hist-8000 simply counts the occurrences of each trigram in the split sequence to build an 8000-d BoW-like representation. 10foldCV: 10-fold Cross-Validation, LR: Logistic Regression, SoT: Sum-of-learnt-Trigrams, Hist-8000: Histogram-8000, AUC: Area Under the Curve, SSL: Self-Supervised learning, BoW: Bag-of-Words, 8000-d: 8000-dimensional.

The Sum-of-learnt-Trigrams (SoT) representation (or ProtVec [17]) utilises the 100-d trigram embeddings derived from self-supervised learning (word2vec Skip-gram [12]) on a large dataset of experimentally-verified sequences (Swiss-Prot database [41]). Word2vec aims to learn continuous distributed trigram representations based on the context they are found within the protein sequences, and as a consequence, trigrams with similar biochemical characteristics are grouped together in the resultant embedding space [17]. By splitting each protein in shifted overlapping trigrams, the pre-trained trigram embeddings are summed up to obtain a 100-d protein representation. See Fig 1 and the cited papers above here for more details on this representation.

Further to the word2vec-based SoT representation, we looked at the powerful zero-shot ProtT5 embeddings model from the more recent ProtTrans work [35]. At downstream inference time, ProtT5 protein embeddings are obtained after a forward pass of the input protein sequence through the encoder of a pre-trained T5 network [42] and then averaging the final hidden layer output embeddings for each amino acid. More specifically, we use here the best-performing fine-tuned transformer model from the ProtTrans work which was “T5-XL”, and was pre-trained on the UniProt50 dataset of 45 million unlabelled protein sequences [41] in a self-supervised learning fashion. That is, the pre-training objective is to predict the missing word in between of the context around it in an auto-encoding fashion (masked language modelling task), which was shown to be necessary over auto-regressive approaches (next-word prediction task) to improve downstream performance of the embeddings [35].

Additionally, we re-purpose the Bag-of-Words (BoW) method from Natural Language Processing and Computer Vision [13] that is often used to construct image vectors from low-level image features and applied it to protein sequences (Fig 1). We term this approach as Histogram-8000 (Hist-8000). It involves splitting each protein sequence into shifted overlapping trigrams, counting the occurrences of each trigram, and then constructing an 8000-d histogram as our protein representation.

### Protein inference problems

The protein representation methods described in this study were tested on twelve protein inference tasks:

- 1. Antigen [43,44]: Antigens are defined as bacterial proteins that can “lead to significant protection (p < 0.05) in an animal model following immunisation and subsequent challenge with the bacterial pathogen” [43]. We use the carefully-curated BPAD200 dataset of 200 antigen and 200 non-antigen proteins [43].
- 2. Enzyme identification [45]: Enzymes are abundant proteins that act as catalysts to eanable and speed up many chemical reactions within the cell. They are usually specific to certain types of reactions, and are especially involved in metabolic processes. The binary data we use consists of: enzyme proteins from the EC-numbers EXPASY-ENZYME database of hierarchical categorisation of enzymes which is linked to Swiss-Prot entries [46], and non-enzyme proteins sampled from Swiss-Prot as described before [45].
- 3. Adhesin identification [47]: An adhesin is a protein, usually from a pathogen, that can attach to the surface of host cells (e.g. human). This step is often part of pathogenicity i.e. the process under which pathogens cause disease to host organisms.
- 4. Virulence Factor [48] (VF): Proteins that enable pathogens to infect hosts and contribute to the pathogen’s ability to cause disease. They are hierarchically categorised and could include antigen or adhesin proteins amongst other functional subsets.
- 5. Allergen [49]: Often foreign to the host, these proteins trigger a strong immune response to a perceived threat that would otherwise be harmless. This results in triggering various undesirable reactions and symptoms. Identifying allergen proteins can be important for designing vaccines that are not harmful to the host organism.
- Sub-cellular localisation (6. gram- bacteria; 7. gram+ bacteria; 8. archaeal) prediction [50]: Predicting which cellular region of the cell a protein would most likely end up in. Here we aim to discriminate between non-cytoplasmic (incl. membrane) and cytoplasmic proteins in the bacterial and archaeal domains of life. Gram+ bacteria lack an additional outer cell membrane in contrast with gram- bacteria.
- TAPE tasks [15]: We sought to further validate our results by evaluating the protein representations on the Tasks Assessing Protein Embeddings benchmark (TAPE) [15]. This consisted of: 9. remote homology (large multi-class classification of protein folds); 10. fluorescence; 11. stability (the latter are both protein engineering regression tasks).
- 12. Family classification [17]: Inspired from the original ProtVec paper, here we set binary classification tasks for the top-25 most frequently occurring protein families in the Swiss-Prot dataset [51], where the proteins of each family have some evolutionary relation and generally similar functions. The top-5 of those are: 50S ribosome-binding GTPase; Helicase conserved C-terminal domain; ATP synthase alpha-beta family (nucleotide-binding domain); 7-transmembrane receptor (G protein-coupled receptor)-rhodopsin family; Amino acid kinase family. In the main text, for brevity we are showing the results for the top-5 families by number of proteins, see S1 File (supporting information) section ‘Simple Bag-of-Words outperforms Sum-of-learnt-Trigrams representations for protein inference’ where we provide the full experiment on the top-25 families in Swiss-Prot. Comparing against these tasks from the original ProtVec study helps establish further the significance and consistency of our results trends.

See the related papers for more details on each task’s dataset, negative samples selection, pre-processing etc. We believe that this wide range of biological challenges will evaluate thoroughly how well the protein representations capture & understand distinct properties of proteins, which is desirable for general-purpose representations [17]. Such representations have previously been successful in language modelling [52]. Moreover, the size of our datasets spanned over four degrees of magnitude and diverse types of species including many microbial proteins not frequently studied with language models, all of which aid in deriving more broad conclusions about our representations.

### Experimental setup

The classification setup is kept as consistent as possible across the protein representations on all inference tasks (Fig 1). In all cases, only proteins containing the 20 standard amino acids [53] are utilised, which yield 8000 possible amino acid trigrams for the Sum-of-learnt-Trigrams (SoT) and Histogram-8000 (Hist-8000) representations. The classifier implemented for each task is a simple Logistic Regressor without any hyper-parameter tuning. For seven tasks (1, 3-8), we also experiment with more classifiers combined with hyper-parameter tuning, to help ensure that the results reflect the effectiveness of the representations, not of any other variable in the process (results section ‘Simple Bag-of-Words still matches Sum-of-learnt-Trigrams representations for protein inference, when evaluated under varying 1 experimental setup settings’). The classifiers implemented in this case are: Logistic Regression, Random Forest, Support Vector Machine (linear & non-linear). In all problems & experiments though, we use the Area Under the Curve (AUC) metric for evaluating the generalisation performance of each classifier model, unless stated otherwise. Where possible, AUC scores were obtained by 10-fold Cross-Validation, with stratified 90-10% train-test splits and shuffling of the data-points prior to splitting to aid successful model training.

Feature selection was conducted on the Hist-8000 representations to identify the most important features (trigram bins) in discriminating between the two classes in each of eight tasks (1-8). Per dataset, this includes: (a) Keeping only the first 2000 trigrams by most occurrences across the proteins; (b) ranking the features remaining in terms of their Fisher ratio [54]; (c) Select the top-200 features as the most informative (200 features were sufficient to build classifiers giving high AUC scores ∼=100% in all tasks); (d) Repeat (a)-(c) through bootstrapping with replacement [54] to arrive at 50 sets of top features. In the end, we can rank individual features based on how often they are found in these 50 sets, arriving at N final top features selected for each task dataset. Thus, we can incrementally build and evaluate reduced Histogram representations using the final top selected N features (i.e Histogram-N, N < 8000). Furthermore, we can carefully analyse the final top selected trigram features of the histogram representations for enrichment in domain compared to non-domain regions of protein sequences, across each inference function problem.

Reducing the amino acid alphabet of the proteins provided another way to build more efficient histogram representations, and in the past has led to improved protein modelling results [55]. In this work we have used the SDM12 (Structural Derived Matrix-12) alphabet of 12 groups of amino acids from [56], which is based on building phylogenetic trees, clustering amino acids and designing substitution matrices derived from structural alignments of proteins with low sequence identity. This was the top-performing method in a large-scale comparison of different amino acid alphabet schemes by [57] and results in a 1728-d Histogram representation which we term Hist-SDM12 (Histogram-Structural Derived Matrix-12).

In addition to the above, we compute pairwise similarity scores on 10 sets of 1000 randomly sampled proteins from the Swiss-Prot database [41]. Cosine similarity scores are computed from the representations, and Needleman-Wunch alignment scores (NW) from the sequences, akin to previous work [18,58]. NW served as the ground truth as it is based on the evolutionary understanding of proteins [59]. Hence, we aim to see which representations are closer to the ground truth, by computing the Spearman rank correlation (*ρ*) between the cosine similarity scores and those from NW. *ρ* enables us to compare scores that are based on different scales.

## Results

### Simple Bag-of-Words outperforms Sum-of-learnt-Trigrams representations for protein inference

**Fig 2:**
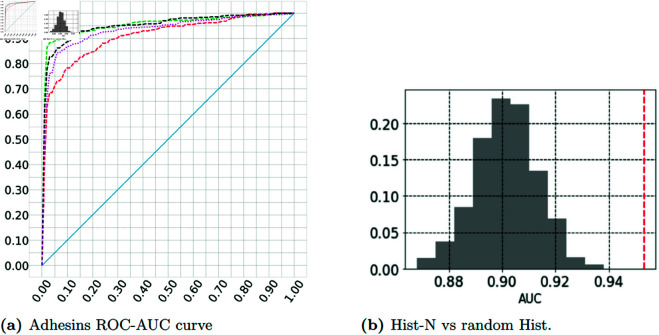
Histogram representations outperform Sum-of-learnt-Trigrams representations in protein inference. Both (a) and (b) are for the adhesins data and LR classifier, see S1 File (supporting information) section ‘Simple Bag-of-Words outperforms Sum-of-learnt-Trigrams representations for protein inference’ for rest of tasks where the trend is largely the same. See (a) for the mean ROC-AUC curves after 10foldCV. In (b), the Hist-N representation (vertical dotted line) is highly accurate (scores found after the 90th percentile) when compared to the distribution of AUCs from 1000 random feature sets. In (a), x-axis is FPR and y-axis is TPR. In (b), y-axis is frequency of score. See section ‘Protein inference problems’ for data sources. Green curve: Hist-8000, red: SoT, black: Hist-N, purple: Hist-SDM12, blue: random classifier. Hist-8000: Histogram-8000, SoT: Sum-of-learnt-Trigrams, ROC: Receiver Operator Characteristic curve, AUC: Area Under the Curve, 10foldCV: 10-fold Cross-Validation, FPR: False Positive Rate, TPR: True Positive Rate, Hist-N: Histogram-N (selected features), Hist-SDM12: Histogram-Structural Derived Matrix-12, LR: Logistic Regression, S1: S1 File (supporting information).

From the twelve protein inference tasks in this work, the Histogram-8000 (Hist-8000) method for representing proteins matches the Sum-of-learnt-Trigrams (SoT) method in all tasks, except only for the Virulence Factors (VFs) task. In certain cases Hist-8000 outperforms the State-Of-The-Art model for the task, however this was not the primary purpose of the experiment. In tasks 1-8, by using a subset of the top features derived via feature selection to build Hist-N (Histogram-N) representations, we are able to find optimal N features that improve upon or match the logistic regression classification performance of SoT in all tasks (apart from VFs). The same outcome compared to SoT is observed for the case of the more biologically meaningful Hist-SDM12 reduced representations (Histogram-Structural Derived Matrix-12), which are also more efficient than the full Hist-8000. See Fig 2 for the logistic regression 10-fold cross-validation classification Receiver-Operator-Characteristic Area-Under-the-Curve results for the adhesins task which illustrates an example of the overall prevalence of Hist-8000, Tables 1 and 2, and the S1 File (supporting information) for the rest of the inference tasks when using logistic regression (S1 File section ‘Simple Bag-of-Words outperforms Sum-of-learnt-Trigrams representations for protein inference’).

**Table 1:** 10-Fold Cross-Validation Area-Under-the-Curve scores (mean ± st.dev.) of protein representation methods in the inference tasks. Hist-8000 outperforms SoT in seven out of tasks 1-8. Hist-8000 consists of the conceptually simpler BoW approach [13], in contrast to SoT which requires SSL pre-training (word2vec) on a large protein sequence dataset [17]. Best-performing methods in bold. See section ‘Protein inference problems’ for task data sources. Gram-negative, Gram-positive and Archaea datasets are each used for subcellular localisation prediction from protein sequence. Gram-neg.: Gram-negative bacteria, Gram-pos.: Gram-positive bacteria, #Proteins (pos.+neg.): number of proteins (positive+negative), Hist-8000: Histogram-8000, BoW: Bag-of-Words, SoT: Sum-of-learnt-Trigrams, Hist-N: Histogram-N (N=number of top selected features), Hist-SDM12: Histogram-Structural Derived Matrix-12, SSL: Self-Supervised Learning, VFs: Virulence Factors, st.dev: standard deviation.

Task	#Proteins (pos. + neg.)	SoT	Hist-8000	Hist-N	Hist-SDM12
Antigens [43]	395 = 196 + 199	0.581 ± 0.080	0.731 ± 0.050	**0.811 ± 0.040**	0.623 ± 0.049
Enzymes [45]	212313 = 74007 + 138306	0.776 ± 0.003	**0.837 ± 0.003**	0.756 ± 0.004	0.802 ± 0.003
Adhesins [47]	1172 = 469 + 703	0.910 ± 0.023	**0.957 ± 0.023**	0.953 ± 0.016	0.936 ± 0.025
VFs [48]	8482 = 3572 + 4910	**0.703 ± 0.016**	0.633 ± 0.021	0.651 ± 0.016	0.661 ± 0.014
Allergenicity [49]	20139 = 10064 + 10075	0.890 ± 0.007	**0.988 ± 0.002**	0.911 ± 0.006	0.966 ± 0.004
Gram-neg. [50]	8205 = 5014 + 3191	0.965 ± 0.007	**0.987 ± 0.003**	0.957 ± 0.008	0.973 ± 0.004
Gram-pos. [50]	2639 = 1816 + 823	0.954 ± 0.013	**0.972 ± 0.015**	0.970 ± 0.014	0.954 ± 0.016
Archaea [50]	802 = 670 + 132	0.966 ± 0.023	**0.978 ± 0.016**	0.974 ± 0.010	0.972 ± 0.024

**Table 2:** 10-Fold Cross-Validation Area-Under-the-Curve scores (mean ± st.dev.) of protein representation methods in five protein family inference tasks. Hist-8000 (results in bold) outperforms SoT in identifying proteins from all the 25 families tested here from the ProtVec study [17]. We note that for each set of protein sequences belonging to a family to be predicted, we randomly sampled the same number of sequences from other families from Swiss-Prot [41] to form the negative data for a balanced task dataset. For brevity we are showing the results for the top-5 families by number of proteins, see S1 File (supporting information) section ‘Simple Bag-of-Words outperforms Sum-of-learnt-Trigrams representations for protein inference’ for the full results on all 25 families. Hist-8000: Histogram-8000, SoT: Sum-of-learnt-Trigrams, st.dev: standard deviation, #Proteins: number of proteins, nt: nucleotide.

Task	#Proteins	SoT	Hist-8000
50S ribosome-binding GTPase	6162	0.982 ± 0.003	**0.993 ± 0.004**
Helicase conserved C-terminal domain	5030	0.963 ± 0.005	**0.992 ± 0.003**
ATP synthase alpha/beta family (nt-binding domain)	4738	0.993 ± 0.004	**0.997 ± 0.003**
7-transmembrane receptor	3600	0.975 ± 0.008	**0.994 ± 0.004**
Amino acid kinase family	3500	0.960 ± 0.009	**0.992 ± 0.004**

The scores obtained from Hist-N are compared to those from 1000 Histogram representations with randomly chosen N features, akin to previous work [44]. The same classification setup is used as above, with a 10-fold cross-validation over a logistic regression classifier. Across all tasks 1-8, the features selected for Hist-N are statistically the best-performing set of features, with Area Under the Curve scores found after the 90th percentile of the distribution of random scores. See Fig 2 for these results for the adhesins task, and the S1 File for the rest (S1 File section ‘Simple Bag-of-Words outperforms Sum-of-learnt-Trigrams representations for protein inference’).

Moreover, the Spearman rank correlation (*ρ*) of Needleman-Wunch sequence alignment (NW) with the Histogram representations cosine similarity scores is higher than that of NW with SoT scores. Hence, the conceptually simpler Hist-8000 and Hist-SDM12 capture the true rank of the sequence similarity scores better than SoT. In Table 3 we provide the mean correlation scores with std.dev. from the 10 iterations of each representation.

**Table 3:** Hist-8000 sequence similarity scores are closer to the ground truth than the corresponding Sum-of-learnt-Trigrams scores. ρ(NW, Hist-8000) and ρ(NW, Hist-SDM12) are both higher than ρ(NW,SoT) for 10 sets of 1000 proteins sampled from Swiss-Prot [41]. ρ close to 1 indicates a strong positive relationship between the alignment and cos. similarity scores, while values closer to zero indicate no relationship. We report mean and standard deviation of the correlations. NW: Needleman-Wunch global sequence alignment algorithm, Hist-8000: Histogram-8000, SoT: Sum-of-learnt-Trigrams, ρ: Spearman rank correlation, Hist-SDM12: Hist-SDM12: Histogram-Structural Derived Matrix-12.

Method	ρ with NW scores
Hist-8000	0.744±0.009
SoT	0.473±0.030
Hist-SDM12	0.724±0.012

### Comparison of Histogram-8000 representation to ProtT5 embeddings

We aim to further evaluate the Histogram-8000 (Hist-8000) representations by comparing them to a more recent embedding approach, namely ProtT5 [35]. In this case, the Hist-8000 representations match the performance of the ProtT5 method in 4 out of the 7 function inference problems 1, 3-8 considered (except antigens, adhesins, virulence factors). Note that each task dataset here is reduced to roughly 90% per their original size, due to computational complexity issues with representing sequences longer than 1000 amino acids when using the ProtT5 method. Also, note that we do not test the ProtT5 method on the enzymes task because of the large size of the dataset that again made it computationally challenging to build the representations. The same classification setup is followed as for the comparison between Hist-8000 vs Sum-of-learnt-Trigrams (section: ‘Simple Bag-of-Words outperforms Sum-of-learnt-Trigrams representations for protein inference’), with a Logistic Regression classifier used here for the ProtT5 method, whereas for Hist-8000 we use for comparison the top-performing classifier model after the experimental evaluation tuning process described in section ‘Simple Bag-of-Words still matches Sum-of-learnt-Trigrams representations for protein inference, when evaluated under varying experimental setup settings’. See Table 4 for an overview of the results.

**Table 4:** 10-fold Cross-Validation Area-Under-the-Curve scores (mean ± st.dev.) of ProtT5 vs Histogram-8000 protein representation methods in the inference tasks. Hist-8000 matches ProtT5 in four out of seven tasks compared (tasks 1, 3-8). Hist-8000 consists of the conceptually simpler BoW approach [13], in contrast to ProtT5 which requires SSL pre-training of a transformer T5 model with three billion parameters on a dataset of 45 million sequences [35]. Best-performing methods in bold. See section ‘Protein inference problems’ for task data sources. Hist-8000: Histogram-8000, BoW: Bag-of-Words, SoT: Sum-of-learnt-Trigrams, SSL: Self-Supervised Learning, VFs: Virulence Factors, Gram-pos: Gram-positive, Gram-neg: Gram-negative, st.dev: standard deviation.

Inference Task	Dataset: number of proteins (positive + negative)	ProtT5	Hist-8000
Antigens [43]	395 = 196 + 199	0.790±0.073	0.747±0.068
Adhesins [47]	1172 = 469 + 703	0.991±0.005	0.958±0.021
VFs [48]	8482 = 3572 + 4910	0.916±0.009	0.724±0.008
Allergenicity [49]	20139 = 10064 + 10075	0.993±0.001	0.991±0.001
Cellular localisation [50]			
Gram-negative bacteria	8205 = 5014 + 3191	0.997±0.001	0.988±0.003
Gram-positive bacteria	2639 = 1816 + 823	0.991±0.006	0.981±0.008
Archaea	802 = 670 + 132	0.994±0.006	0.980±0.012

### Simple Bag-of-Words still matches Sum-of-learnt-Trigrams representations for protein inference, when evaluated under varying experimental setup settings

We also sought to evaluate the Histogram-8000 (Hist-8000) and SoT (Sum-of-learnt-Trigrams) representation methods under different classifier models and hyper-parameter combinations. For tasks 1, 3-8, we thoroughly test the representations using the following three classification models: Logistic Regression (LR), Random Forest (RF), Support Vector Machine (SVM). This choice of models ensures that we are testing the effect of linear classifiers compared to non-linear ones (including kernel SVMs). We also experiment with a range of hyper-parameter combinations to cover the space of model configurations that could affect the performance in identifying proteins. Those consist of 10 hyper-parameter configurations for LR, 30 for SVM and 27 for RF classifiers. The main outcome is for most problems similar to the initial experiment in section ‘Simple Bag-of-Words outperforms Sum-of-learnt-Trigrams representations for protein inference’, i.e. that Hist-8000 still matches the SoT method in six out of seven problems as shown in Table 5. An exception was the virulence factors problem where SoT was better than Hist-8000 by about 2% C.V. AUC. This outcome confirms that the choice of protein sequence representation is the most important step in predicting protein functions. Here we can observe that the top-performing models for each of SoT and Hist-8000 were closer in terms of mean scores in each problem. SoT representations particularly show a slightly improved performance when coupled with a non-linear RF classifier (virulence factors, archaeal localisation, allergen identification problems). As we can observe in other sections with protein inference results, for smaller datasets we obtain higher standard-deviation of prediction scores during cross-validation which indicates that models tend to be more confident when trained on more protein sequences, as expected.

**Table 5:** 10-fold Cross-Validation Area-Under-the-Curve scores (mean ± st.dev.) of protein representation methods in the inference tasks, under varying experimental setup settings. Hist-8000 matches SoT in six out of seven tasks (tasks 1, 3-8 considered here), after thorough comparisons of the representation methods under different classifier models and their hyper-parameters. Hist-8000 consists of the conceptually simpler BoW approach [13], in contrast to SoT which requires SSL pre-training (word2vec) on a large protein sequence dataset of more than 500k proteins [17]. Best-performing representations in bold, with the classifier used for the top representation method provided in separate column. See section ‘Protein inference problems’ for task data sources. Hist-8000: Histogram-8000, BoW: Bag-of-Words, SoT: Sum-of-learnt-Trigrams, AUC: Area Under the Curve, SSL: Self-Supervised Learning, LR: Logistic Regression, RF: Random Forest, VFs: Virulence Factors, Gram-pos: Gram-positive, Gram-neg: Gram-negative, st.dev: standard deviation, #Proteins: number of proteins.

Inference Task	#Proteins (pos. + neg.)	Classifier	SoLT	Hist-8000
Antigens [43]	395 = 196 + 199	LR	0.705±0.062	0.747±0.068
Adhesins [47]	1172 = 469 + 703	LR	0.934±0.024	0.958±0.021
VFs [48]	8482 = 3572 + 4910	RF	0.747±0.017	0.724±0.008
Allergenicity [49]	20139 = 10064 + 10075	RF	0.992±0.001	0.991±0.001
Cellular localisation [50]				
Gram-negative bacteria	8205 = 5014 + 3191	LR	0.984±0.002	0.988±0.003
Gram-positive bacteria	2639 = 1816 + 823	RF	0.979±0.006	0.981±0.008
Archaea	802 = 670 + 132	RF	0.985±0.010	0.980±0.012

### Analysis of Histogram-8000 representation per protein region (domain vs non-domain)

When analysing the top N features selected (N < 8000) for the reduced Histogram-N (Hist-N) representations per task (tasks 1-8), we can find the protein regions (domain vs non-domain) in which these features are over-represented, in total across the datasets. For each protein, the region occurrences of the trigram feature in question are divided by the length of each region to enable a consistent comparison between domain and non-domain region coverage by the feature. Then, we sum up the coverage percentages of the feature across the dataset sequences. This leaves us with 2 total coverage numbers (domain vs non-domain) per feature, from which we can see where the feature is enriched across the task dataset. Repeating this process for all features would reveal whether most top features from Hist-N are enriched in domain or non-domain regions (Table 6). Also, to quantify further the importance of domain and non-domain regions in capturing the properties of proteins, we conduct the same classification experiment (10-fold Cross-Validation – Logistic Regression), but with the Histogram-8000 (Hist-8000) representations built with amino acids from only the two respective regions each time (domain vs non-domain). Overall, there is consistency between which region is found to be enriched with the top Hist-8000 features per task and which Hist-8000 region-specific representations infer properties better, between domain and non-domain regions (Table 6). This suggested that Hist-8000 captures most of the required information to classify the proteins correctly in a data-driven way, without specifying a-priori which regions are most important. We have six of tasks 1-8 pointing to this, except for allergens and virulence factors (the latter being the only case where Sum-of-learnt-Trigrams was better than Hist-8000 in the experiments). In the case of the antigenicity problem, there is some recent literature supporting the unconventional result that the functional signal is enriched in non-domain features, specifically intrinsically disordered regions [60,61].

**Table 6:** 10-fold Cross-Validation Area-Under-the-Curve scores (mean ± st.dev.) of domain vs non-domain representations in the protein inference tasks. Overall, there is consistency between region enrichment of the top Hist-8000 features selected and best-performing Hist-8000 region-specific representations, between domain and non-domain regions per task (tasks 1-8). Best-performing methods in bold. See section ‘Protein inference problems’ for tasks sources. Hist-8000: Histogram-8000, SoT: Sum-of-learnt-Trigrams, AUC: Area Under the Curve, 10foldCV: 10-fold Cross-Validation, VFs: Virulence Factors, Gram-pos: Gram-positive, Gram-neg: Gram-negative, st.dev: standard deviation, doms: domains, non-doms: non-domains, #Top feats: number of top features.

Task	Hist-8000 (doms)	Hist-8000 (non-doms)	#Top feats (doms — non-doms)
Antigens [43]	0.564±0.081	0.627±0.102	1720 (851 — 869)
Enzymes [45]	0.849±0.003	0.685±0.004	533 ( 282 — 251)
Adhesins [47]	0.968±0.012	0.889±0.016	1056 ( 543 — 513)
VFs [48]	0.612±0.025	0.543±0.016	68 (10 — 58)
Allergenicity [49]	0.990±0.002	0.972±0.003	550 (234 — 316)
Cellular localisation [50]			
Gram-negative bacteria	0.978±0.004	0.927±0.009	582 ( 372 — 210)
Gram-positive bacteria	0.974±0.009	0.932±0.029	977 ( 631 — 346)
Archaea	0.966±0.027	0.925±0.051	1454 ( 856 — 598)

Despite that all task datasets have >30% of 1-domain proteins (see S1 File section ‘Exploring domain-based models for function inference’), it’s still reasonable to see Hist-8000 features enriched in domain regions in five problems, as we have at least ∼40% of proteins in each of the 8 datasets covered by domains for over 70% of their sequence. Finally, we note that for this part we work only with proteins having both at least one domain and non-domain region to enable the region-specific analysis, which means that a small part (∼10% ) of each dataset is left out. We think this has not affected the conclusions made further on, as the main trend of Hist-8000 (over the whole sequence) being the best method has remained for tasks 1-8, despite a small difference in some scores (See S1 File section ‘Analysis of representations per protein region (domain vs non-domain)’).

### TAPE benchmark results

We sought to further validate our results by evaluating the protein representations on the TAPE benchmark (Tasks Assessing Protein Embeddings) [15]. This consists of the remote homology (large multi-class classification of protein folds), fluorescence and stability tasks (the latter are both protein engineering regression tasks). We again use Logistic Regression to carry out classification for the remote homology problem, and a standard Linear Regressor for the protein engineering problems. For each dataset, only a single train-test set split is curated in the TAPE paper [15] given certain biological constraints, which limits our ability to quantify the generalisation of the models to the extent done for the other inference tasks in this work.

Similarly to all other results, the Histogram-8000 (Hist-8000) method for representing proteins outperforms Sum-of-learnt-Trigrams (SoT) in all three protein inference datasets from the TAPE benchmark. In addition, Hist-8000 was more effective than all baseline representations and some powerful neural network model representations that were not pre-trained, however the primary purpose of the experiment is not to compete with the State-Of-The-Art. It is worth noting that the low performance results on the remote homology problem observed across all representation methods are due to the challenging nature of predicting a protein fold from just the sequence, which means overcoming a gap of two evolutionary distance levels on the SCOP hierarchy of structural information [62]. On top of that, there are 1195 fold classes to learn to predict which necessarily makes this problem quite challenging to model. See Table 7 for more details on SoT vs Hist-8000 on the TAPE tasks.

**Table 7:** Prediction scores of protein representations in the TAPE inference tasks. Hist-8000 outperforms SoT in all three tasks. The One-hot representation approach is based the occurrences of the 20 amino-acids in sequence, and was used as baseline in the TAPE study [15]. The neural network architectures (Transformer, ResNet, LSTM) did not go through a pre-training phase. Spearman rank correlation ρ is used as a metric to assess method performance in the stability and fluorescence tasks, and the standard accuracy metric for the remote homology problem. Best-performing methods in bold. Hist-8000: Histogram-8000, SoT: Sum-of-learnt-Trigrams, ρ: Spearman rank correlation, NNs: Neural Networks, ResNet: Residual neural Network, LSTM: Long-Short-Term-Memory recurrent neural network, TAPE: Tasks Assessing Protein Embeddings benchmark [15].

Task	Hist-8000	SoT	One-hot	Transformer	ResNet	LSTM
Remote Homology (Accuracy)	$\underset{―}{0.10}$	0.07	0.09	0.09	0.10	0.12
Stability (Spearman *ρ*)	$\underset{―}{0.47}$	0.30	0.19	–0.06	0.61	0.28
Fluorescence (Spearman *ρ*)	$\underset{―}{0.48}$	0.40	0.14	–0.22	–0.28	0.21

We note that for this set of tasks we were unable to carry out region-enrichment analysis of any informative histogram features selected, because of the kind of protein functions studied. More specifically, the remote homology problem consists of domain sequences, except for 66 multi-domain sequences which is a small fraction of the whole dataset. Regarding the two regression problems (stability, fluorescence), in each there is a main protein from which the rest of the sequences are generated by mutating that main sequence to study changes in its function, thus we would only have meaningful domain region information for those two initial proteins. Also, uncertainty estimates are not calculated because of the difficulty of constructing multiple data splittings brought by the biological characteristics of the protein engineering tasks (for example the mutation distance-based train-test sets design, where we would possibly need more informative ground-truth labels to construct meaningful evaluations).

## Discussion

In this study, we show that baseline Bag-of-Words (BoW) inspired representations systematically match ProtT5 and Sum-of-learnt-Trigrams (SoT) representations in a range of protein inference tasks and in encoding sequence similarity. It was hypothesised that Self-Supervised-Learning (SSL) models would emulate the success seen in Natural Language Processing (NLP) [16], as the pre-training process attempted to extract biologically meaningful features by learning continuous distributed representations for sequence sub-units based on the context in which they appear in many experimentally-annotated protein sequences. In contrast, Histogram-8000 (Hist-8000) is a conceptually simple statistical method based on building a bag of trigram counts in the protein sequence and hence ignoring any evolutionary patterns present. The result of a simpler method eliminating the need for more complicated ones has been observed before in other inference problems within the broader field of machine learning [63].

The trend of Hist-8000 and its related reduced representations (Histogram-N, Histogram-Structural Derived Matrix-12) performing better than the dense 100-d SoT on protein classification tasks opposes what is stated by the Curse of Dimensionality [64]. Because of the high sparsity in the input data distribution induced by this representation, one might expect that training downstream classifiers that generalise well on unseen data becomes more challenging. However, sparse data representations have shown promising performance in several problems such as image processing [65], which might mean that the representations manage to express the intrinsic structure of the data suitably for some of the problems studied here. Given the classification results, it is not surprising to see Hist-8000 encoding more information about protein sequence similarity than the SoT method, as sequence similarity is a minor but useful factor for determining several protein properties [4]. Importantly, the fact that SoT is worse than the sequence alignment baseline for remote homology detection defeats the purpose of considering SoT as an efficient alternative method for overcoming the limitations of alignment, more specifically in identifying the structural fold of a protein when sequence similarity is low. Favourable results for representations based on BoW were obtained recently in related fields [13,66–69]. Thus, it can be said that Hist-8000 removes the need for self-supervised learning pre-training for obtaining meaningful sub-unit (i.e. biological word) embeddings and subsequently protein representations. Even by considering the superior performance of ProtT5 in some of the function inference tasks, using Hist-8000 would save computational resources from the most expensive part of building large language model representations: that is, pre-training on approx. 45 million sequences, learning 3 billion parameters, and requiring expensive processing hardware. This pre-training process required over 10 hrs per epoch and at least 26 GBs of memory in the ProtTrans study [35]. However, we note the high time complexity observed when using the Hist-8000 representations during downstream inference, which we can estimate to be 80-times slower than SoT and 8-times slower than ProtT5 embeddings given their respective dimensions. Given the potential for more efficient sequence-based representations for challenging problems such as identifying virulence factors [48], it would be beneficial to re-examine the SoT and other language-based machine learning approaches for building dense representations that are more biologically accurate across problems, without high computational requirements.

We could partially attribute the lower performance of the protein representation methods in certain problems to the choice of biological words (trigrams, amino acids). It has been documented that there is no universal criterion for how the protein sequence should be split for further representation modelling [13]. We note in particular a couple of studies where summing the elements in the 100-d SoT representations to just 1 number (namely ProtVec1D vectors) produced almost identical results to using the whole 100-d sequence vectors, which are already a summation of the 100-d trigram vectors obtained from pre-training [24,25]. Future work could include approaches such as protein domain embeddings [70] as a more biologically inspired choice of “words” and way of splitting the protein sequences, which would also lessen the effect of the method used to combine words to form the protein embedding, since we would have much fewer words per protein than when using trigrams. On the other hand, the success of those models depends on the distribution of domains in the data as mentioned before [70–72], and which is evident from our prototype experiment where the high number of 1-domain proteins seem to negatively affect the classification scores (S1 File section ‘Exploring domain-based models for function inference’).

Throughout most of the literature on representation learning applied to protein sequences, it has been assumed that the standard 20 amino acids alphabet is the most suitable choice for the biological alphabet [13]. However, for certain protein inference tasks, it was demonstrated that it is beneficial to consider reduced-sized amino acid alphabets by grouping the standard amino acids based on biologically inspired criteria [55]. This future research direction also opens up the possibility of data-driven and task-specific tuning of representations. By reducing the amino acid alphabet used for building embeddings, one can model longer n-grams which would result in capturing longer discriminative amino acid motifs and also in reduced features sparsity with models running on less memory in turn [13].

Finally, another shortcoming of the SoT method is the use of model configurations for word2vec that were selected with NLP tasks in mind, for learning the biological word representations. This has been observed across several studies that used SoT representations [17,23,28–30] and in work applying newer language models for representing biological sequences for inference problems [73,74]. These model configurations include mathematical approximations, for example linear hidden layer activation functions or negative sampling, the latter which is a main component of word2vec [12] to deal with the large number of weight updates dictated by the input vocabulary size. In contrast, the available datasets with experimentally-derived protein sequences yield smaller vocabularies and corpora. That is still true when using domains as sequence sub-units, where we approximately have 15k different domains [70,72] compared to the over ∼500k words found in english. Thus, as future work it could be important to quantify protein inference performance when using representations derived from predecessor NLP models that can be trained without the aforementioned approximations [75].

## Supporting information

S1 File Supporting information document. (PDF)
