# Supplementary material for: Bag-of-words is competitive with sum-of-embeddings language-inspired representations on protein inference
Source: PLoS One. 2025 Aug 6;20(8):e0325531. doi: 10.1371/journal.pone.0325531 (PMC12327643; doi:10.1371/journal.pone.0325531)
Supplement: S1 File — (PDF) [file pone.0325531.s001.pdf]

Bag-of-words is competitive with sum-of-embeddings language-inspired representations on protein inference

Frixos Papadopoulos[1*], Tilman Sanchez-Elsner[2], Mahesan Niranjan[1], Ashley I. Heinson[3]

1 Vision-Learning-Control group, Department of Electronics and Computer Science, Faculty of Engineering and Physical Sciences, University of Southampton, Southampton, United Kingdom. **2** Clinical and Experimental Sciences, Department of Medicine, University of Southampton, Southampton, United Kingdom. **3** Clinical Informatics Research Unit, Cancer Sciences, Department of Medicine, University of Southampton, Southampton, United Kingdom

* fp1n17@soton.ac.uk (FP)

Supporting Information

Exploring domain-based models for function inference

Continuing on from the domain enrichment analysis for the Histogram-8000 (Hist-8000) representations (main text section 'Analysis of Histogram-8000 representation per protein region (domain vs non-domain)'), to test whether domain-based models can improve the function inference results, we construct a Sum-of-Domains (SoD) representation and applied it to the function tasks 1-8. The domain embeddings come from earlier work on applying word2vec on protein pseudo-sentences made up of protein domains (pfam [1]), with the goal of utilising the distribution of contexts in which each domain is found in, in order to capture its biological meaning in the mathematical representation [2]. The word2vec model used is very similar in terms of hyper-parameters to the work done in the original Sum-of-learnt-Trigrams (SoT) representation paper (ProtVec [3]), even though in the original study the sequence sub-units were modeled as triplets of amino acids instead. Our experiment here carries on from previous domain-based analysis and extends the domain embeddings evaluation to carry out inference of global protein properties from sequence information, akin to other work [4]. The same classification setup is followed as with the other representations in the experiment in the main text section 'Simple

Bag-of-Words outperforms Sum-of-learnt-Trigrams representations for protein inference', with 10-fold Cross-Validation over a Logistic Regression classifier (for more see main text section: 'Materials and methods'), but with the balanced accuracy metric for evaluation.

Across all 16 function prediction tasks (excluding the three TAPE benchmark tasks where this does not apply), we find that over 30% of the proteins in each dataset have only 1 pfam domain region found in their sequence. In the case of the enzymes task for example, this is quite major, with 50% of proteins having 1 domain. It has been documented previously that the number of available domain annotations outside of the eukaryotic kingdom, which is where most of our inference data lies in, is comparatively lower [2, 5]. This suggested that the overlaps between representations of different proteins could be high depending on the domains distribution, which we link to the classification results next.

The SoD classification results compared to SoT and Hist-8000 are generally lower across tasks 1-8). For the domain-based SoD models, we notice these trends quantified via Spearman's correlation ρ (Table 1): (i) achieving lower classification scores when the percentage of proteins in the whole dataset with only 1 sequence domain is higher [ρ(SoD_scores,%-1domain-proteins)=-0.623, p-val=0.099)], and (ii) achieving lower classification scores when the percentage of domains that appear in protein(s) in both classes is higher [ρ(SoD_scores,%-domains-both-classes)=-0.524, p-val=0.183)]. After investigating, we also see that this coincides and could be attributed to a sizeable portion of the proteins having 1-domain duplicates between the two classes, which would make it difficult for any classifier based on SoD representations to learn discriminating accurately the proteins (Table 1). Indeed, we subsequently observe that 1-domain proteins with identical representations are frequently mis-classified across some problems, for example with the antigens problem we have ~53.8% of 1-domain proteins being mis-classified (see Table 1). In case we do not include the aforementioned proteins with identical domains which cause the representations duplication issue in the data, then we would lose a sizeable amount of proteins from training the function prediction models effectively.

Finally, we note that for this part we work only with proteins having at least one domain in their sequence, which means that a small part (~10%) of each dataset is left out. We believe this does not impact the comparisons made between the different kinds of protein representations in this work or the analysis results.

Task	Number of Proteins	Num. of distinct Domains (pfam)	SoD 10foldC.V. score	(%) of 1-domain **proteins** in data (out of all proteins in task data)	(%) of **domains** that are in both prot. classes (out of all domains in task)	(%) of 1-domain **proteins** -> which have the same SoD representation with >=1 protein(s) in other class (out of all proteins in task)	(%) of 1-domain proteins with identical protein(s) in other class -> which were misclassified
Antigens	395	434	0.545 ± 0.046	45.3%	11.5%	6.6%	53.8% (14/26)
Enzymes	212625	14823	0.701 ± 0.005	51.6%	18.8%	15.9%	31.2% (10579/33900)
Adhesins	1172	988	0.894 ± 0.046	45.3%	0.3%	0%	0%
VFs	8482	3248	0.687 ± 0.020	49.4%	19.4%	15.4%	16.6% (216/1302)
Allergenicity	20139	5651	0.864 ± 0.005	56.7%	5.1%	17.1%	9.3% (319/3436)
Cellular localisation							
Gram-neg. bacteria	8205	1981	0.912 ± 0.008	33.4%	7.8%	1.3%	27.4% (29/106)
Gram-pos. bacteria	2639	989	0.906 ± 0.018	32.5%	5.9%	1.0%	18.5% (5/27)
Archaea	802	312	0.903 ± 0.058	31.9%	0.6%	0%	0%

Table 1. Sum-of-Domains representations function prediction performance is hindered by the distribution of sequence domains. Overall, SoD scores lower than other representations in this work (for tasks 1-8). This can be partially attributed to the high number of 1-domain protein duplicate representations and their between-class overlap across the datasets which contributes to considerable mis-classifications. See main text section 'Protein inference problems' for task data sources. Metric: balanced accuracy. SoD: Sum-of-Domains, VFs: Virulence Factors, Gram-pos: Gram-positive, Gram-neg: Gram-negative, 10foldCV: 10-fold Cross-Validation.

Simple Bag-of-Words outperforms Sum-of-learnt-Trigrams representations for protein inference

Fig 1 displays the full ROC-AUC (Receiver-Operator-Characteristic Area-Under-the-Curve) results from the classification tasks 1-8 of the experiments in this work.

Fig 2 displays the full classification results on the comparison of: the top Histogram features mathematically selected per task, to 1000 random representations (tasks 1-8). For the enzymes task, we use only 250 random representations because of computational complexity issues.

Fig 3 displays the full ROC-AUC results for the top-5 most frequent protein families for the families classification tasks borrowed from the ProtVec paper [3].

Table 2 displays the results for all the 25 protein families used to test the representations thoroughly on the families classification tasks borrowed from the ProtVec paper [3].

Analysis of representations per protein region (domain vs non-domain)

Table 3 displays the full (ROC-AUC) results from the classification tasks 1-8 for the SoT (Sum-of-learnt-Trigrams) and Hist-8000 (Histogram-8000) representations built based on three different sequence parts: whole sequence; domain regions only; non-domain regions only. In this way, we aim to quantify further the importance of domain and non-domain regions in capturing the properties of proteins. The classification setup remains the same (10foldCV, Logistic Regression, ROC-AUC). As mentioned in main text section 'Analysis of Histogram-8000 representation per protein region (domain vs non-domain)', for this part we work only with proteins having both at least one domain and non-domain

Inference Task (Asgari & Mofrad 2015)	**Dataset:** number of proteins (positive + negative)	SoT	Hist-8000
50S ribosome-binding GTPase	6162 (3081+3081)	0.982 ± 0.003	**0.993 ± 0.004**
Helicase conserved C-terminal domain	5030 (2515+2515)	0.963 ± 0.005	**0.992 ± 0.003**
ATP synthase alpha/beta family (nucleotide-binding domain)	4738 (2369+2369)	0.993 ± 0.004	**0.997 ± 0.003**
7-transmembrane receptor	3600 (1800+1800)	0.975 ± 0.008	**0.994 ± 0.004**
Amino acid kinase family	3500 (1750+1750)	0.960 ± 0.009	**0.992 ± 0.004**
ATPase family associated with various cellular activities	3420 (1710+1710)	0.968 ± 0.009	**0.994 ± 0.005**
tRNA synthetases class I	3266 (1633+1633)	0.994 ± 0.005	**0.998 ± 0.003**
tRNA synthetases class II	2830 (1415+1415)	0.916 ± 0.014	**0.992 ± 0.005**
Major Facilitator Superfamily	2604 (1302+1302)	0.983 ± 0.006	**0.991 ± 0.004**
Hsp70 protein	2532 (1266+1266)	0.990 ± 0.006	**0.998 ± 0.002**
Histidine biosynthesis protein	2496 (1248+1248)	0.986 ± 0.005	**0.996 ± 0.003**
NADH-Ubiquinone-plastoquinone (complex I), various chains	2478 (1239+1239)	0.988 ± 0.004	**0.993 ± 0.005**
TCP-1-cpn60 chaperonin family	2476 (1238+1238)	0.979 ± 0.010	**0.987 ± 0.007**
EPSP synthase (3-phosphoshikimate 1-carboxyvinyltransferase)	2408 (1204+1204)	0.987 ± 0.005	**0.997 ± 0.004**
Aldehyde dehydrogenase family	2392 (1196+1196)	0.980 ± 0.007	**0.997 ± 0.003**
Shikimate–quinate 5-dehydrogenase	2256 (1128+1128)	0.937 ± 0.016	**0.995 ± 0.005**
GHMP kinases N terminal domain	2236 (1118+1118)	0.943 ± 0.006	**0.987 ± 0.003**
Ribosomal protein S2	2166 (1083+1083)	0.985 ± 0.007	**0.993 ± 0.005**
Ribosomal protein S4–S9 N-terminal domain	2136 (1068+1068)	0.993 ± 0.005	**0.999 ± 0.003**
Ribosomal protein L16p-L10e	2106 (1053+1053)	0.984 ± 0.009	**0.987 ± 0.008**
KOW motif	2092 (1046+1046)	0.979 ± 0.007	**0.996 ± 0.003**
Uncharacterized protein family UPF0004	2086 (1043+1043)	0.990 ± 0.004	**0.996 ± 0.004**
Ribosomal protein S12-S23	2024 (1012+1012)	0.993 ± 0.006	**0.993 ± 0.006**
GHMP kinases C terminal	2022 (1011+1011)	0.959 ± 0.011	**0.993 ± 0.005**
Ribosomal protein S14p-S29e	1994 (997+997)	0.982 ± 0.008	**0.995 ± 0.005**

Table 2. 10-fold Cross-Validation Area-Under-the-Curve scores (mean ± st.dev.) of protein representation methods in 25 protein family inference tasks. Hist-8000 (results in bold) outperforms SoT in identifying proteins from all the 25 families tested here from the Asgari and Mofrad 2015 study [3]. Hist-8000: Histogram-8000, SoT: Sum-of-learnt-Trigrams, st.dev: standard deviation.

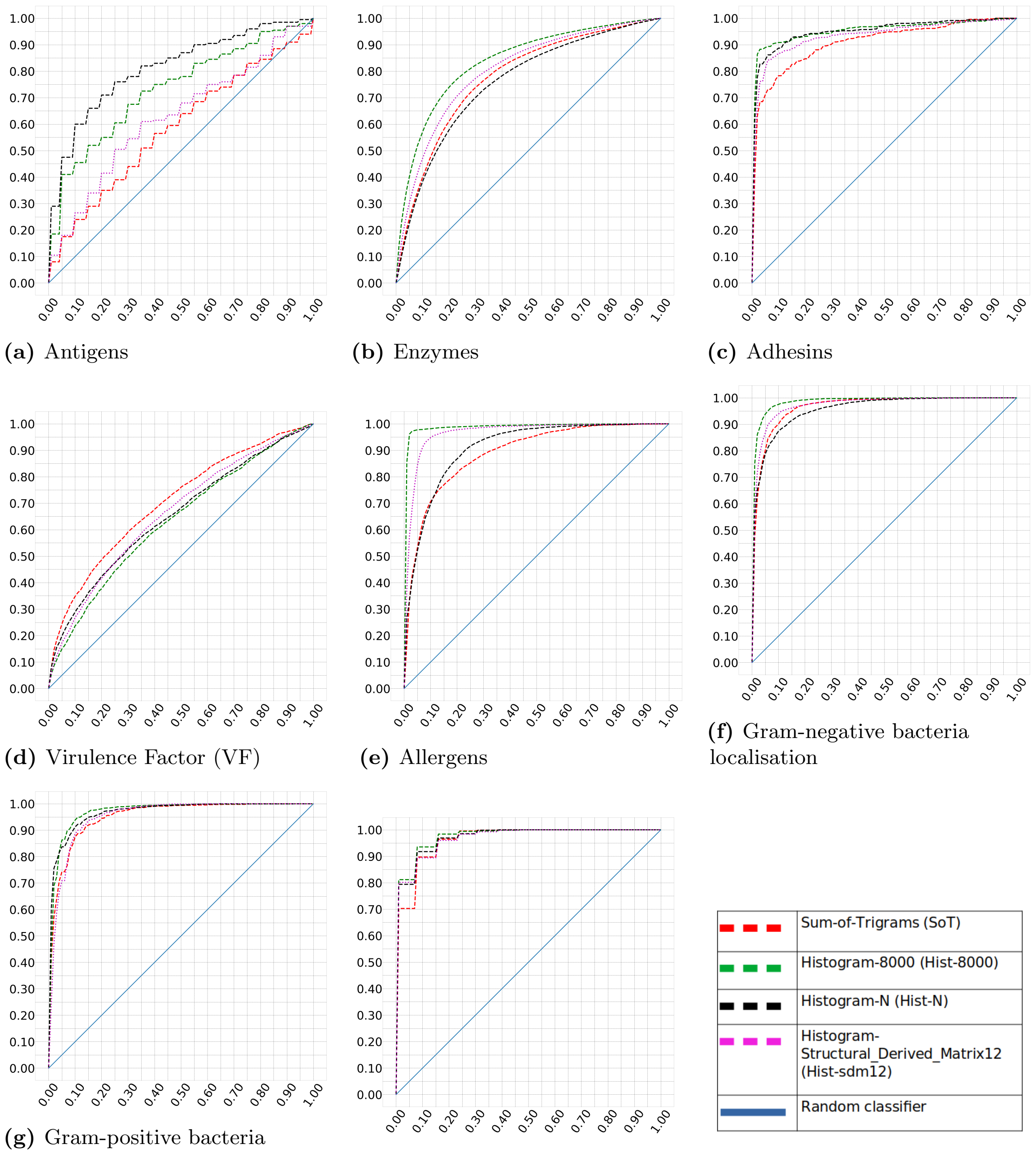

(a) Antigens **(b)** Enzymes **(c)** Adhesins **(d)** Virulence Factor (VF) **(e)** Allergens **(f)** Gram-negative bacteria localisation **(g)** Gram-positive bacteria localisation **(h)** Archaeal localisation **(i)** Legend

Fig 1. Histogram representations outperform Sum-of-learnt-Trigrams representations in protein inference tasks. ROC-AUC curves for each representation method are after 10foldCV over each task's classifier (tasks 1-8). x-axis is FPR and y-axis is TPR. ROC: Receiver Operator Characteristic curve, AUC: Area Under the Curve, 10foldCV: 10-fold Cross-Validation, FPR: False Positive Rate, TPR: True Positive Rate.

region, which means that a small part (~10%) of each dataset was left out. We think this has not affected the conclusions made further on, as again in this comparison the main trend of Hist-8000 (over the whole sequence) being the best method has remained for tasks 1-8, despite a small difference in some

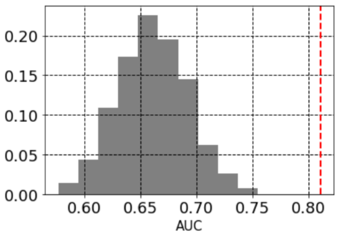

(a) Antigens

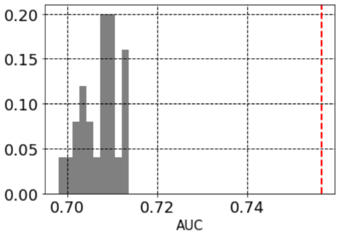

(b) Enzymes

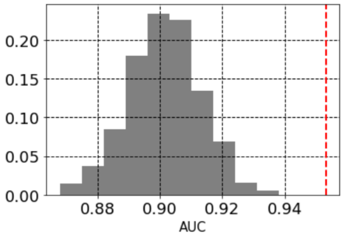

(c) Adhesins

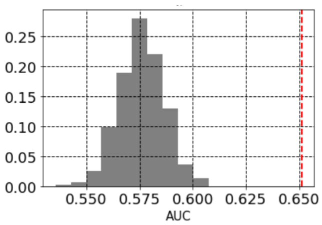

(d) Virulence Factor (VF)

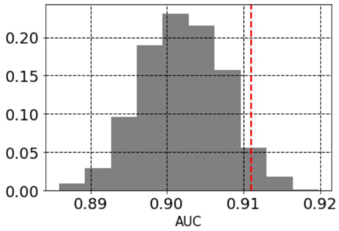

(e) Allergens

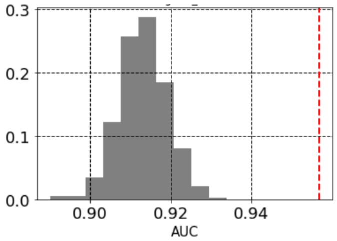

(f) Gram-negative bacteria localisation

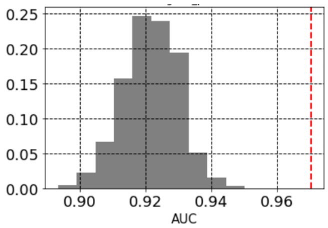

(g) Gram-positive bacteria localisation

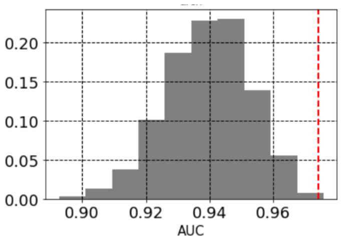

(h) Archaeal localisation

Fig 2. Scores from Histogram representations with selected features are statistically significant compared to the distribution of scores from random representations. Across tasks 1-8, the Hist-N representation of selected features (vertical dotted line) is highly accurate (scores found after the 90th percentile) when compared to the distribution of AUCs from random feature sets, with Hist-N classification scores. y-axis is frequency of score. AUC: Area Under the Curve, Hist-N: Histogram-N (selected features).

scores. Moreover, in six out of eight inference tasks, there is consistency between which region-specific representations perform better and the regions we find the most informative features, following the feature selection process outlined in main text section 'Experimental setup'.

Task	Hist-8000	SoT	Hist-8000 (dom)	Hist-8000 (n.d.)	SoT (dom)	SoT (n.d.)	Top features enriched in regions:
Antigens	**0.677 ±0.066**	0.640 ±0.035	0.564 ±0.081	0.627 ±0.102	0.618 ±0.074	0.532 ±0.090	Non-domain
Enzymes	0.832 ±0.004	0.767 ±0.003	**0.849 ±0.003**	0.685 ±0.004	0.782 ±0.003	0.655 ±0.003	Domain
Adhesins	**0.976 ±0.014**	0.942 ±0.013	0.968 ±0.012	0.889 ±0.016	0.919 ±0.020	0.874 ±0.020	Domain
VFs	0.608 ±0.021	**0.670 ±0.015**	0.612 ±0.025	0.543 ±0.016	0.655 ±0.019	0.600 ±0.023	Non-domain
Allergens	**0.990 ±0.002**	0.892 ±0.006	**0.990 ±0.002**	0.972 ±0.003	0.886 ±0.007	0.812 ±0.008	Non-domain
Cellular localisation							
Gram-neg. bacteria	**0.986 ±0.004**	0.960 ±0.006	0.978 ±0.004	0.927 ±0.009	0.952 ±0.007	0.883 ±0.008	Domain
Gram-pos. bacteria	**0.980 ±0.008**	0.958 ±0.013	0.974 ±0.009	0.932± 0.029	0.926 ±0.014	0.901 ±0.033	Domain
Archaea	**0.985 ±0.011**	0.969 ±0.018	0.966 ±0.027	0.925 ±0.051	0.946 ±0.032	0.822 ±0.072	Domain

Table 3. 10-fold Cross-Validation Area-Under-the-Curve scores (mean ± st.dev.) of whole sequence vs domain vs non-domain representations in the inference tasks. Overall, Hist-8000 representations, either from the full sequence or just the domain regions, once again perform best across the tasks 1-8. Best-performing methods in bold. See main text section 'Protein inference problems' for task data sources. Hist-8000: Histogram-8000, SoT: Sum-of-learnt-Trigrams, AUC: Area Under the Curve, VFs: Virulence Factors, Gram-pos: Gram-positive, Gram-neg: Gram-negative, n.d: non-domain region, dom: domain region, Hist-N: Histogram-N (selected features).

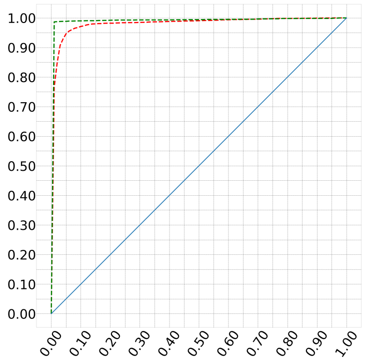

(a) 50S ribosome-binding GTPase

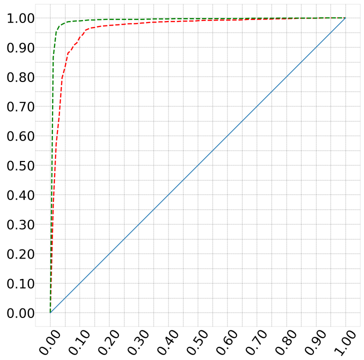

(b) Helicase conserved C-terminal domain

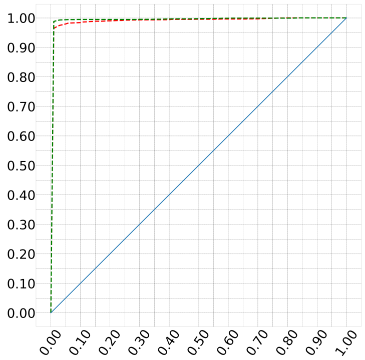

(c) ATP synthase alpha/beta family, nucleotide-binding domain

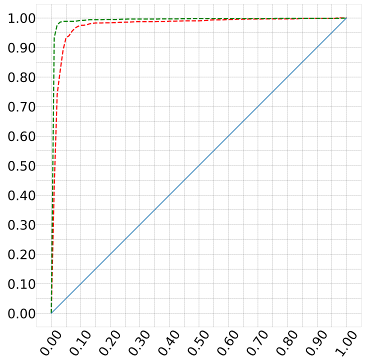

(d) 7 transmembrane receptor (rhodopsin family)

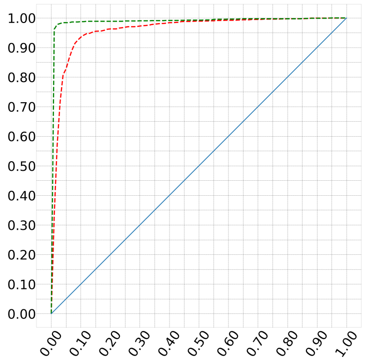

(e) Amino acid kinase family

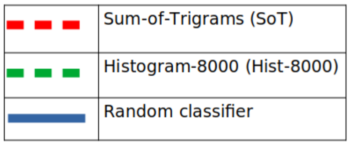

(f) Legend

Fig 3. Histogram representations outperform Sum-of-learnt-Trigrams representations in protein family inference tasks. ROC-AUC curves for each representation method are after 10foldCV over each task's classifier. Here we show plots of five of the 25 family prediction tasks results for brevity. x-axis is FPR and y-axis is TPR. ROC: Receiver Operator Characteristic curve, AUC: Area Under the Curve, 10foldCV: 10-fold Cross-Validation, FPR: False Positive Rate, TPR: True Positive Rate.

References

1. Mistry J, Chuguransky S, Williams L, Qureshi M, Salazar GA, Sonnhammer ELL, et al. Pfam: The protein families database in 2021. Nucleic Acids Research. 2021;doi:10.1093/nar/gkaa913.

2. Buchan DWA, Jones DT. Learning a functional grammar of protein domains using natural language word embedding techniques. Proteins: Structure, Function, and Bioinformatics. 2020;doi:10.1002/prot.25842.

3. Asgari E, Mofrad MRK. Continuous distributed representation of biological sequences for deep proteomics and genomics. PLoS ONE. 2015;doi:10.1371/journal.pone.0141287.

4. Melidis DP, Nejdl W. Capturing Protein Domain Structure and Function Using Self-Supervision on Domain Architectures. MDPI Algorithms. 2021;doi:10.3390/a14010028.

5. Williamson MP. How Proteins Work. Garland Science; 2012.
